# Establishment and Application of Real-Time Fluorescence Quantitative PCR Detection Technology for *Metschnikowia bicuspidata* Disease in *Eriocheir sinensis*

**DOI:** 10.3390/jof9080791

**Published:** 2023-07-27

**Authors:** Yuenan Xing, Ye Chen, Chengcheng Feng, Jie Bao, Xiaodong Li, Hongbo Jiang

**Affiliations:** Aquaculture Department, College of Animal Science and Veterinary Medicine, Shenyang Agricultural University, Shenyang 110866, China; 2007500011@syau.edu.cn (Y.X.); 2020240613@stu.syau.edu.cn (Y.C.); 2018500015@syau.edu.cn (C.F.); lixiaodong@syau.edu.cn (X.L.)

**Keywords:** *Eriocheir sinensis*, *Metschnikowia bicuspidata*, milky disease, mitochondrial cytochrome c oxidase subunit VIA, qPCR

## Abstract

*Metschnikowia bicuspidata* causes a “milky disease” in Chinese mitten crab, *Eriocheir sinensis*, which inflicts significant damage on the breeding industry, but there are no effective drugs for this disease. Precise detection technologies and clarification of transmission routes are now essential to prevent its occurrence. A real-time fluorescent quantitative PCR (qPCR) detection method targeting the mitochondrial cytochrome c oxidase subunit VIA (COX6A) of *M. bicuspidata* was developed and its sensitivity, specificity, repeatability, and application effectiveness evaluated. There was a robust linear relationship between the qPCR threshold cycle value (Ct) and copy number of the standard with a wide dynamic range. The standard curve had a correlation coefficient (R^2^) of 0.996, amplification efficiency of 103.092%, and a lower limit of detection sensitivity of 7.6 × 10^1^ copies/µL. The COX6A-qPCR method exhibited high specificity for the detection of *M. bicuspidata*, with no cross-reactivity. The intra- and inter-group variation coefficients were <1% and 2%, respectively. The qPCR exhibited superior sensitivity compared to existing detection methods, with a positivity rate of 76.67%. The *M. bicuspidata* content ranged from 1.0 × 10^1^–2.7 × 10^6^ copies/µL. The COX6A-qPCR detection technology exhibited high sensitivity, strong specificity, and excellent repeatability, enabling the accurate quantification of *M. bicuspidata*.

## 1. Introduction

*Metschnikowia bicuspidata* was initially isolated from diseased *Daphnia magna* in 1884 [1], and there are currently three reported variants: *M. bicuspidata* var. Bicuspidata, *M. bicuspidata* var. California, and *M. bicuspidata* var. Chathamia [2,3]. *Metschnikowia bicuspidata* is an opportunistic pathogen that can infect various economically important aquatic animals, such as *Eriocheir sinensis*, *Macrobrachium rosenbergii*, *Palaemonetes sinensis*, *Portunus trituberculatus*, and *Oncorhyncus tshawytscha*. Their presence can thus seriously hinder healthy development in aquaculture systems [3,4,5,6,7,8,9,10].

Chinese mitten crabs, *Eriocheir sinensis*, when infected with *M. bicuspidata*, exhibit symptoms including swollen joints, white muscles, and milky hemolymph, which is why it is also referred to as “milky disease”. Following infection, the vitality of crabs decreases, and their appendages become prone to detachment, ultimately leading to death due to extensive yeast infections throughout the body [11]. Since 2019, outbreaks of “milky disease” in many Chinese mitten crab breeding areas have resulted in significant economic losses to the aquaculture industry.

The current commonly employed detection method is conventional PCR technology, with primers designed for the identification of yeast 26S rDNA and 5.8S ITS rDNA gene sequences [11,12]. However, these methods exhibit low specificity and sensitivity, which may lead to false positive results owing to cross-reactivity with other similar aquatic animal diseases. However, the accurate diagnosis of crabs with mild initial infection levels is hindered by low sensitivity, thereby impeding disease prevention and treatment efforts. The previously reported nested PCR detection technology, which is highly sensitive and specific, is time-consuming and does not enable the direct quantification of *M. bicuspidata* in Chinese mitten crabs [13].

Real-time quantitative PCR (qPCR) is a modified version of conventional PCR that utilizes fluorescent probes to detect and quantify unknown templates through the accumulation of fluorescence signals [14]. Commonly employed methods for the quantitative detection of yeast fluorescence include both dye and probe approaches. The TaqMan probe method uses an oligonucleotide segment that can hybridize and complement a target gene. Chen et al. [15] established a qPCR identification method for *Saccharomyces cerevisiae*, *Pichia spartinae*, *Zygosaccharomyces rouxii*, and *Dekkera bruxellensis* by targeting a conserved sequence of the yeast gene. Liu et al. [16] developed a detection method for *M. bicuspidata* utilizing the TaqMan probe method; however, the design of the probe proved challenging, and its synthesis cost exorbitant, resulting in a high level of expense. The SYBR Green I fluorescence method is a cost-effective and user-friendly nonspecific minor groove binder that intercalates with the DNA double helix, making it a widely applicable approach for disease detection.

In this study, we aim to develop a SYBR Green I qPCR method that exhibits robust specificity and high sensitivity in detecting *M. bicuspidata*, thereby providing valuable assistance for the prevention and treatment of milky disease in Chinese mitten crabs.

## 2. Materials and Methods

### 2.1. DNA Extraction from Chinese Mitten Crabs

Chinese mitten crabs were obtained from a breeding farm in Panjin City, Liaoning Province and were subsequently transported to the laboratory at Shenyang Agricultural University. DNA was extracted from the hepatopancreatic tissues using a marine animal tissue DNA extraction kit (Tiangen [Beijing] Biochemical Technology Co., Ltd., Beijing, China). The extracted DNA was quantified using an ultramicro spectrophotometer (K5500, Beijing Kaiao Technology Development Co., Ltd. Beijing, China) and diluted to 100 ng/µL before being stored at −20 °C.

### 2.2. Test Strains

*M. bicuspidata* (LNES0119), which was preserved in our laboratory, was cultivated in Bengal Red culture medium for activation. After being cultured at 28 °C for 48 h, a single colony was selected and streaked again on Bengal red culture medium. Subsequently, a single colony was used for DNA extraction and stored at −20 °C. *Metschnikowia pulcherrima*, *Wicherhamomyces anomalus*, *Staphylococcus aureus*, *Bacillus lateralis*, white spot syndrome virus, and *Enterocytozoon hepatopenaei* were preserved in our laboratory for primer-specific detection.

### 2.3. Establishment of a qPCR Detection System for M. bicuspidata

#### 2.3.1. Primer Design and Synthesis

Based on the reference gene sequence for *M. bicuspidata* published in NCBI, the mitochondrial cytochrome c oxidase subunit VIA (COX6A) gene (gene number: XM_018858497.1) was used as a template for qPCR with specific primers P1/P2, designed using Primer 5.0 software. Conventional PCR primers were designed based on the 26S rDNA and 5.8S-ITS rDNA sequences of *M. bicuspidata* [11,12]. The previously published outer primers PN1/PN2 and inner primers PN3/PN4, which were designed based on the HYR cell wall protein gene, were used as primers for the nested PCR [13] (Table 1).

#### 2.3.2. Conventional and Nested PCR Reaction Procedures and Systems

The standard PCR reaction procedure was as follows: initial denaturation at 95 °C for 10 min, followed by denaturation at 95 °C for 1 min, annealing at 55 °C for 45 s, and extension at 72 °C for 1 min, for 35 cycles, and then the final extension at 72 °C for an additional 10 min. The conventional PCR system comprises 25 µL, and it contains 13 µL of 2 × Taq Master Mix, 10 µL of ddH_2_O, and 0.5 µL each of the upstream and downstream primers, with an additional 1 µL of DNA template.

The nested PCR reaction protocol involves pre-denaturation at 95 °C for 10 min, followed by denaturation at 95 °C for 1 min, annealing at 55 °C for 45 s, and extension at 72 °C for 1 min, and this cycle is repeated 35 times, followed by a final extension step at 72 °C for an additional 10 min. The nested PCR reaction system is 25 μL, and it contains 12 μL of 2 × Taq Master Mix, 10 μL of ddH_2_O, and 0.5 µL each of the upstream and downstream primers, along with 2 μL of the DNA template. The second round of nested PCR amplification employed the same reaction system as the first round, with the sample DNA for this round diluted by a factor of 1000 from the product obtained in the initial amplification.

#### 2.3.3. Preparation of Plasmid Standards

For target gene amplification, PCR amplification with fluorescent quantitative primers P1/P2 (Table 1) was performed using the positive DNA for *M. bicuspidata* as the template. The reaction protocol involved an initial denaturation step at 95 °C for 5 min, followed by 35 cycles of denaturation at 95 °C for 30 s, annealing at 60 °C for 30 s, and extension at 72 °C for 30 s. The final extension was performed at 72 °C for 7 min. The total reaction volume comprised a mixture containing two times Taq Master Mix (12.5 µL), ddH_2_O (8 μL), upstream primer (1 µL), downstream primer (1 µL), and DNA template (2.5 µL).

For the purification and recovery of DNA fragments, 10 μL of PCR products were subjected to electrophoresis on a 1.5% agarose gel containing nucleic acid dye for 30 min (120 V). Following electrophoresis, the target band at 140 bp was excised from the gel with precision. Subsequently, the gel fragment containing the desired band was placed in a centrifuge tube and DNA extraction was performed using the FastPure Gel Mini Kit (Vazyme, Nanjing, China) for efficient recovery.

For vector recombination, a reaction solution, which was 5 µL, was prepared by combining the pMD-19-T vector (1 µL), extracted DNA sample (1 µL), and ddH_2_O (3 µL). Subsequently, Solution I (5 µL) was added to the prepared reaction solution and incubated overnight at 4 °C.

For strain screening, the carrier solution was combined with *Escherichia coli* DH5 α. The recipient cells were then added to a 1.5 mL centrifuge tube, placed on ice for 30 min, then heated to 42 °C, 890 µL of Luria Bertani (LB) medium was added, shaken well at 37 °C, and the selector plate was prepared using LB agar medium containing ampicillin (AMP)-β-D thiopyranogalactoside supplemented with 40 µL X-gal and 5 µL isopropyl. Subsequently, a bacterial solution (sterilized water:1:1) was applied to the selection board. The board was then sealed and placed in a 37 °C incubator overnight. Afterward, a white colony from the plate was scraped with an inoculum and dissolved in 100 µL ddH_2_O. Finally, a plasmid DNA sample of 5 μL was taken for validation by sequencing (Sangon, Shanghai, China). The remaining liquid was transferred to a centrifuge tube containing 5 mL of AMP-LB medium and incubated in a shaker at 37 °C for approximately 4 h until the solution became turbid with mycelia. Subsequently, plasmids were extracted using the FastPure Plasmid Mini Kit (Vazyme, Nanjing, China).

#### 2.3.4. Establishment of qPCR Reaction Conditions

The successfully sequenced recombinant plasmid was selected as the standard after measuring the initial concentration and calculating the copy number. Subsequently, standard plasmids were diluted 10-fold. A gradient of recombinant plasmids ranging from 10^7^ to 10^1^ copies/μL was selected as the standard, with a reaction system consisting of 20 μL of ddH_2_O replacing the DNA sample in the blank group (Table 2). Multiple gradient experiments were performed to optimize the primer concentration, adjust the annealing and extension temperatures, and determine the optimal conditions for qPCR amplification.

#### 2.3.5. qPCR Standard Curve

The recombinant plasmid was serially diluted 10 times to generate seven standard samples with concentrations ranging from 10^7^ to 10^1^ copies/µL. A standard curve was constructed to correlate the logarithm (x) of the initial number of standard templates with the cyclic threshold number (Ct) of the amplification products, and the correlation coefficients and amplification efficiency were analyzed to determine the qPCR standard curve.

#### 2.3.6. Specific Testing

*M. bicuspidata* was used as a positive control and ddH_2_O as the negative control, while the DNA of *M. pulcherrima*, *W. anomalus*, *S. aureus*, *B. lateralis*, white spot syndrome virus, and *E. hepatopenaei* served as specific identification templates for qPCR detection using an optimized reaction system and conditions.

#### 2.3.7. Sensitivity Testing and Clinical Sample Analysis

The recombinant plasmid standard was serially diluted by a factor of 10 and used as a template for routine PCR amplification with the primer pair P1/P2. The detection limit analysis was performed using 1.5% agarose gel electrophoresis. Similarly, qPCR was performed using the same primer pair, P1/P2, to determine the sensitivity and minimum detection limit of both qPCR and routine PCR. Sixty clinical samples from Chinese mitten crabs were then subjected to qPCR, nested PCR, and conventional PCR.

#### 2.3.8. Repetitive Experiments

To assess the stability of the qPCR, continuously diluted positive plasmid DNA was used as a qPCR template. Three duplicate template concentrations were set for each template to evaluate the stability through intra- and inter-group replication. The mean, standard deviation, and coefficient of variation were calculated using the Ct values repeated thrice within each group [17]. Intergroup replication was conducted by repeating the experiment three times. The mean, standard deviation, and coefficient of variation were computed based on the Ct values obtained from three independent experiments [18].

#### 2.3.9. qPCR Detection of Tissue Tropism and Potential Carriers of *M. bicuspidata*

To assess tissue orientation, three Chinese mitten crabs infected with *M. bicuspidata* were randomly selected, and DNA was extracted from the hepatopancreas, gills, muscles, hemolymph, stomach, heart, eye, ganglion, and intestine for qPCR. Biological samples were collected from rice fields in which Chinese mitten crabs were infected with *M. bicuspidata*. The biological samples consisted of *Chanodichthys erythopterus*, *Pseudorasbora parva*, *Macrobrachium nipponense, Neocaridina denticulata* shrimp, cladocerans, and copepods. DNA extraction was performed using liver tissue from fish, hepatopancreatic tissue from shrimp, and entire tissue samples from cladocerans and copepods. The extracted DNA was diluted to a concentration of 100 ng/µL before qPCR analysis.

## 3. Results

### 3.1. Reaction Conditions for qPCR

The recombinant plasmid’s initial concentration was 23.7 ng/µL, and the copy number was 7.6 × 10^9^ copies/µL. Subsequently, dilution with ddH_2_O was performed continuously ten times to obtain plasmid standard samples ranging from 7.6 × 10^7^–7.6 × 10^1^ copies/µL for qPCR amplification. A 20 µL reaction system was employed using the recombinant plasmid gradient as a reference standard (Table 2), with ddH_2_O replacing the DNA sample in the blank group. After multiple gradient tests, an optimal annealing temperature of 63.5 °C was determined (Table 3). The amplification conditions and the system are presented in Table 2 and Table 3, respectively.

### 3.2. Specific Test

**Figure 1:**
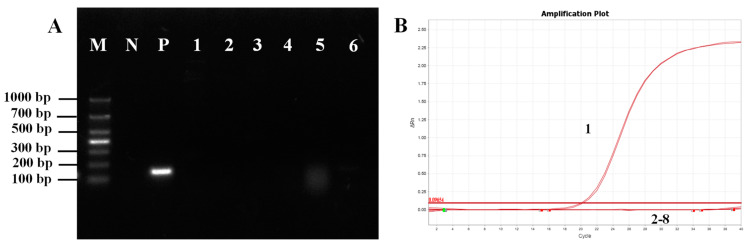
Primer P1/P2 specificity test. (**A**) Routine PCR specificity test. M: Marker; N: Negative control; P: Positive control; 1: *Bacillus lateralis*; 2: *Staphylococcus aureus*; 3: *Enterocytozoon hepatopenaei*; 4: White spot syndrome virus; 5: *Metschnikowia pulcherrima*; 6: *Wicherhamomyces anomalus*. (**B**) Fluorescence quantitative PCR specificity test. 1: Positive control; 2: Negative control; 3: *Bacillus lateralis*; 4: *Staphylococcus aureus*; 5: *Enterocytozoon hepatopenaei*; 6: White spot syndrome virus; 7: *Metschnikowia pulcherrima*; 8: *Wicherhamomyces anomalus*.

Specificity was determined using genetic material from fungi (*M. pulcherrima*, *W. anomalus* and *E. hepatopenaei*), bacteria (*S. aureus* and *B. lateralis*), and viruses (white spot syndrome virus). The results showed that only *M. bicuspidata* yeast exhibited bright and distinct target bands, indicating the excellent specificity of primers P1/P2 (Figure 1A). Furthermore, qPCR was conducted to confirm that the sample contained genetic material from the aforementioned microorganisms, with dd H_2_O serving as the negative control. qPCR was conducted using primers P1/P2, and the amplification curve demonstrated that only samples positive for *M. bicuspidata* exhibited amplification curves. No fluorescence signals were detected for the other microorganisms, further indicating the high specificity of the primer (Figure 1B).

### 3.3. Sensitivity Test

**Figure 2:**
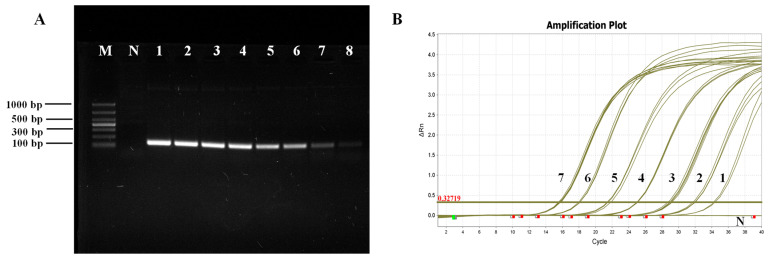
Sensitivity test for primers P1/P2. (**A**) Routine PCR sensitivity test; M: Marker; N: Negative control; 1–8: Plasmid standard 7.6 × 10^9^–7.6 × 10^2^ copies/µL. (**B**) Fluorescence quantitative PCR sensitivity test; N: Negative control; 1–7: Plasmid standard 7.6 × 10^1^–7.6 × 10^7^ copies/µL.

In the standard plasmid template, 7.6 × 10^9^–7.6 × 10^2^ copies/µL were utilized as routine PCR amplification templates, while those ranging from 7.6 × 10^7^–7.6 ×10^1^ copies/µL served as qPCR amplification templates. The PCR amplification product was subjected to electrophoresis on a 1.5% agarose gel, and the detection limit of primer P1/P2 was determined to be 7.6 × 10^2^ copies/µL, with bands appearing faint and barely discernible at this concentration (Figure 2A). The qPCR amplification results showed that fluorescence signals could be detected within the range of 7.6 × 10^7^–7.6 × 10^1^ copies/µL. Even at a template concentration of 7.6 × 10^1^ copies/µL, the amplification curve for fluorescence quantification still exhibited a standard S-curve, with approximately 35 cycles required to reach the threshold, indicating that the detection limit for real-time qPCR was as low as 7.6 × 10^1^ copies/µL (Figure 2B).

### 3.4. qPCR Standard Curve

**Figure 3:**
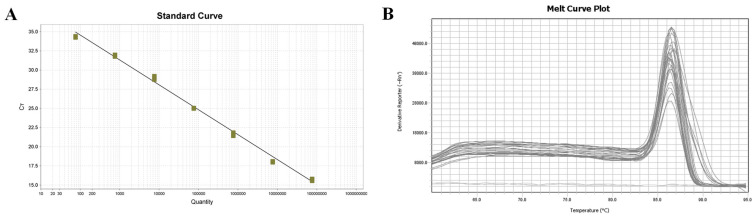
Fluorescence quantitative PCR standard curve and melting curve. (**A**) Standard curve; (**B**) Melting curve.

The fluorescence quantitative amplification curve within the range of the plasmid standard from 7.6 × 10^7^–7.6 × 10^1^ copies/µL more accurately depicts the exponential growth and plateau of the PCR (Figure 3A). The equation for the standard curve is Ct = −3.25log (Sq) + 41.074, with a correlation coefficient of R^2^ = 0.996 and an amplification efficiency of 103.092%. The melting curve analysis (Figure 3B) showed that the curves of three parallel amplification products in each gradient group were merged, resulting in a single melting peak at 86.51 °C, with overlapping positions. This single melting peak indicated that nonspecific amplification or primer dimer formation did not occur during amplification.

### 3.5. Repeatability Testing

Repeatability analysis was conducted on three parallel samples with identical gradients within the group (7.6 × 10^7^–7.6 × 10^1^ copies/µL). The results demonstrated that the coefficient of variation of Ct within the group was <1%, indicating excellent repeatability (Table 4). Moreover, the coefficient of variation between the groups was <2%, further suggesting that this method has good repeatability, which ensures the stability and reliability of the experimental outcomes.

### 3.6. Sample Testing

Routine, nested, and qPCR assays were used to detect DNA extracted from the hepatopancreases of 60 randomly selected Chinese mitten crabs. qPCR analysis revealed that 46 of the 60 Chinese mitten crab hepatopancreatic DNA samples tested positive, with a detection rate of 76.67%. The *M. bicuspidata* content ranged from 1.0 × 10^1^–2.7 × 10^6^ copies/µL. The results of the nested PCR test indicate that 28 samples tested positive in the first round and 39 samples tested positive in the second round, resulting in a detection rate of 65%. In contrast, routine PCR testing, using primers P3/P4, detected only 18 positive samples (30% detection rate), whereas primers P5/P6 detected 29 positive samples (48.33% detection rate). These findings suggest that qPCR is more sensitive than both nested and conventional PCR.

### 3.7. Tissue Tropism and Potential Carriers of M. bicuspidata

The DNA of the hepatopancreas, gills, muscles, hemolymph, stomach, heart, eye, ganglion, and intestine from diseased crabs was extracted for qPCR detection (Table 5). The results indicate that among the tissues taken from the Chinese mitten crabs infected with *M. bicuspidata*, the highest pathogen content was found in the hemolymph, whereas the lowest was detected in the eyes. qPCR was conducted on various biological samples collected from rice paddies, which revealed the presence of *M. bicuspidata* in fish, shrimp, copepods, and cladocerans. Shrimp and Cladoceras exhibited the highest levels (Table 6).

## 4. Discussion

Severe outbreaks of “milky disease” have occurred during the breeding of Chinese mitten crabs in northern China in recent years. The infected crabs exhibited weakened vitality, tissue emulsification, and mortality rates exceeding 20%. Currently, this disease is spreading nationwide, with an increasing annual infection rate. The pathogen responsible for this epidemic is *M. bicuspidata*, for which there are no effective treatments currently available. Zhang et al. [12] discovered that Massoia lactone, produced by *Aureobasidium melanogenum*, exhibits potent anti-*M. bicuspidata* activity. Ma et al. [9] also observed the inhibitory effects of various antifungal drugs, including ketoconazole, fluconazole, econazole, clotrimazole, amphotericin B, itraconazole, and nystatin, against *M. bicuspidata*. However, the clinical efficacy of these drugs is yet to be demonstrated. Therefore, early interruption of the transmission route is the primary approach for preventing disease occurrence. Diseased crabs, in their initial stages, can only be detected using highly sensitive molecular biology techniques because no apparent abnormalities can be observed with the naked eye. Currently, the PCR method commonly used to quantify *M. bicuspidata* lacks accuracy and requires agarose electrophoresis to determine the amplification results, which is a complex and time-consuming process [11,12]. Nested PCR has a higher sensitivity and specificity than conventional PCR; however, its operation is complex and time-consuming. Additionally, it cannot quantify the extent of pathogen infection [13]. qPCR technology possesses not only high sensitivity, good specificity, and accuracy, but also ease of operation and implementation, rendering it widely applicable for the detection of aquatic pathogens [19]. *M. bicuspidata* is an opportunistic pathogen. The establishment of a qPCR detection method enables rapid and precise quantification of *M. bicuspidata* in samples, facilitating accurate diagnosis, even at low levels, during the early stages of the disease outbreak. This is conducive to the prompt implementation of effective prevention and control measures, thereby minimizing economic losses.

In this study, a novel qPCR method was developed to target the mitochondrial cytochrome c oxidase subunit VIA (COX6A) gene in *M. bicuspidata*. The COX6A motif, has a circular DNA molecule located outside the nuclear genome that encodes respiratory chain cytochrome oxidase, and is widely conserved in both prokaryotic and eukaryotic organisms [20]. The utilization of mitochondrial cytochrome C oxidase subunits for species identification has emerged as a new research direction, with many detection methods employing these subunits as target genes. Di Cesare et al. [21] used a specific mitochondrial COX I gene fragment to establish a PCR assay for *Eucoleus boehmi* in canids, whereas Latrofa et al. [22] developed a multiplex PCR assay for filarioid species infesting dogs using mitochondrial COX I. Echeverry et al. [23] established a PCR assay based on mitochondrial COX I to detect Plasmodium parasites, which demonstrated superior sensitivity and specificity compared to 18S rRNA gene-based PCR. The mitochondrial COX6A gene utilized in this study, a subunit of the mitochondrial cytochrome C oxidase complex, exhibits conserved characteristics and low amino acid sequence homology with other yeast species (Table 7). Therefore, mitochondrial COX6A could be used to develop and implement detection technologies for *M. bicuspidata*.

The qPCR technique established in this study exhibited significantly enhanced specificity compared to conventional PCR. The COX6A gene primers demonstrated robust specificity and produced clear bands during sample analysis. Currently, the primers designed for LSU rRNA and its ITS region have the potential to amplify template DNA from other pathogens, resulting in a target band of *Staphylococcus aureus* appearing at the same location as *M. bicuspidata* [13]. The presence of other microorganisms in environmental samples or individuals may result in erroneous detection, and thus necessitating the sequencing of the target band to confirm *M. bicuspidata* identity. The qPCR primers designed for this study exhibited no cross-reactivity with the six other pathogens; thus, any specific amplification confirmed the presence of *M. bicuspidata*, which was validated through clinical sample testing.

The sensitivity of COX6A-qPCR (7.6 × 10^1^ copies/μL) was notably higher than that of the LSU rRNA group (6.03 × 10^4^ copies/μL) and ITS (6.74 × 10^5^ copies/μL) primers (Bao et al., 2022). COX6A-qPCR detected positive results in 46 of 60 clinical samples (76.67%), and sequence alignment analysis confirmed the presence of a COX6A gene fragment specific to *M. bicuspidata*. In contrast, nested PCR yielded a positivity rate of 65%, with 28 positive samples in the first round and an additional 11 positives in the second round. However, in clinical samples, the LSU rRNA and ITS primers only achieved positive rates of amplification products of 30% and 48.33%, respectively, which were significantly lower than those obtained using qPCR. This further confirmed that qPCR exhibited high specificity and sensitivity.

Compared with the other detection methods, qPCR possesses the unique ability to quantify and elucidate the degree of disease in Chinese mitten crabs. The range of yeast content in positive samples calculated in this study is 1.0 × 10^1^–02.7 × 10^6^ copies/µL, indicating that this detection method has a broad detection range and can accurately measure the extent of infection. In this study, the established COX6A-qPCR method was used to detect *M. bicuspidata* in various tissues from the infected Chinese mitten crabs, revealing a significant presence of *M. bicuspidata* across all tissues, with higher yeast content observed in the hemolymph, stomach, hepatopancreas, and muscle than in other tissues. The identification of these tissues could facilitate the detection of positive samples during periods of low *M. bicuspidata* load in Chinese mitten crabs, thereby enhancing detection accuracy and contributing significantly to the early diagnosis, prevention, and control of *M. bicuspidata* disease. The hemolymph of *E. sinensis* was initially tested to determine its infection status. This approach also minimized tissue loss and mortality in the sampled individuals. By utilizing this method for detecting biological samples in the environment, it has been determined that *M. bicuspidata* can be detected in various organisms such as fish, shrimp, copepods, and cladocera, with a higher concentration found within shrimp and cladocera. This suggests that the disinfection of the breeding environment should be conducted beforehand, particularly for environmental organisms such as cladocerans and shrimp, to minimize potential carriers and reduce infection risks.

The qPCR method established in this study exhibited an intragroup coefficient of variation ranging from 0.12–0.51% and an intergroup coefficient of variation ranging from 0.51–1.36%. These results demonstrate that the established method exhibits excellent repeatability. Furthermore, the absence of agarose gel electrophoresis enables the entire detection reaction to be completed within 1.3 h, and real-time fluorescence quantitative amplification curve analysis allows for the interpretation of results in less than half of the time required for conventional and nested PCR. The gene sequences used in this study were derived from a strain of *M. bicuspidata* isolated in the United States, whereas the test samples were obtained from a Chinese mitten crab strain. Despite their disparate origins, these strains exhibited excellent specificity, underscoring the practicality of this detection method.

The qPCR method for *M. bicuspidata* established in this study exhibited high sensitivity, strong specificity, and good repeatability. It could thus be utilized for the rapid and quantitative detection of *M. bicuspidata* in infected individuals as well as for the precise detection of the pathogen in the environment. This is important for elucidating the transmission route of the disease and providing a foundation for accurate prevention and control measures.

## Figures and Tables

**Table 1:** Primer sequences.

Primer Type	Primer Name	Primer Sequence 5′-3′	Fragment Size (bp)
qPCR	P1/P2	P1:ATTCAACTCGCACGGTCAC	140
		P2:TCCACAACTCGGTGATGC	
Conventional PCR	P3/P4	P3:GCATATCAATAAGCGGAGGAAAAG	574
		P4:GGTCCGTGTTTCAAGACGG	
	P5/P6	P5:TCCGTAGGTGAACCTGCGG	394
		P6:TCCTCCGCTTATTGATATGC	
Nested PCR	PN1/PN2	PN1:AGCCTGGTCTTTGTAATG	493
		PN2:ACTCCCTTGTTGGTGATA	
	PN3/PN4	PN3:TTAGAGGGACTTCTCATTTGT	226
		PN4:CTTTAGCGTCAATATCGTAGA	

**Table 2:** qPCR reaction system.

Reaction Composition	Reaction System
2 × ChamQ Universal SYBR qPCR Master Mix	10 μL
Upstream primer	0.4 μL
Downstream primer	0.4 μL
Yeast DNA	2 μL
ddH_2_O	7.2 μL
Total volume	20 μL

**Table 3:** Fluorescence quantitative PCR reaction procedure.

Hold Stage	PCR Stage (40 Cycles)	Melt Curve Stage
95 °C 30 s	95 °C 10 s	95 °C 15 s
	63.5 °C 30 s	60 °C 1 min
	72 °C 30 s	95 °C 15 s

**Table 4:** Repeatability detection of *M. bicuspidata* by COX6A-qPCR.

Template Quantity	Ct Values Intra-Group		Ct Values between Groups	
	Average Value ± Standard Deviation	Coefficient of Variation (%)	Average Value ± Standard Deviation	Coefficient of Variation (%)
7.6 × 10^7^	15.70 ± 0.08	0.51	15.71 ± 0.08	0.51
7.6 × 10^6^	18.05 ± 0.06	0.33	18.04 ± 0.15	0.83
7.6 × 10^5^	21.60 ± 0.07	0.32	21.30 ± 0.29	1.36
7.6 × 10^4^	25.01 ± 0.03	0.12	24.91 ± 0.16	0.64
7.6 × 10^3^	28.89 ± 0.14	0.48	29.05 ± 0.20	0.69
7.6 × 10^2^	31.90 ± 0.13	0.41	31.84 ± 0.17	0.53
7.6 × 10^1^	34.32 ± 0.08	0.23	34.35 ± 0.27	0.79

**Table 5:** Tissue tropism of *M. bicuspidata*.

Tissue	Log (Copies)/100 ng DNA
Hepatopancreas	6.05 ± 0.17
Gill	5.18 ± 0.15
Muscle	6.02 ± 0.23
Hemolymph	6.72 ± 0.21
Stomach	6.52 ± 0.57
Heart	5.07 ± 0.51
Eye	2.40 ± 0.36
Ganglion	5.23 ± 0.26
Intestine	5.24 ± 0.32

**Table 6:** Potential carrier detection.

Species	Number	CT Value	Positive Number	Positive Rate
*Pseudorasbora parva*	6	33.21 ± 0.78	4	66.67%
*Chanodichthys erythropterus*	2	_	0	0
*Macrobrachium nipponense*	4	26.49 ± 0.75	3	75%
*Neocaridina denticulata*	3	31.04 ± 0.55	3	100%
Cladocerans	6	28.91 ± 0.56	6	100%
Copepods	4	24.91 ± 0.27	2	50%

**Table 7:** Multiple sequence alignment analysis of amino acid sequence.

Yeast Species	% Identity	The Accession Number
*Metschnikowia bicuspidata*	–	XM_018858497.1
*Metschnikowia zobellii*	86.75	OW618031.1
*Metschnikowia aff*.	84.03	CP034458.1
*Candida duobushaemulonis*	79.89	XM_025481247.1
*Candida haemuloni var. vulneris*	79.89	CP076662.1
*Candida pseudohaemulonii*	79.26	XM_024857054.1
*Millerozyma farinosa*	76.92	FO082059.1

## Data Availability

All data included in this study are available upon request by contacting the corresponding author.
